# Does Life Lose Its Meaning When the Heart Fails? Illness Perception, Perceived Stress and Meaning in Life in Polish Patients with Heart Failure

**DOI:** 10.3390/healthcare14131889

**Published:** 2026-06-29

**Authors:** Rafał Gerymski

**Affiliations:** Department of Psychology of Individual Differences, Institute of Psychology, Opole University, 45-040 Opole, Poland; rafal.gerymski@uni.opole.pl

**Keywords:** heart failure, meaning in life, illness perception, perceived stress, cardiovascular disease, psychocardiology, health psychology, structural equation modelling, Polish cardiac patients

## Abstract

**Background/Objectives**: Heart failure (HF) is a highly unpredictable disease that significantly impacts patients’ well-being. One of the fundamental problems faced by cardiac patients is trying to answer the question of how to lead a meaningful life. Meaning in life is a crucial predictor of well-being, ill-being and quality of life for everyone, not just cardiac patients. Therefore, identifying its predictors is crucial. Based on Leventhal et al.’s common-sense model of self-regulation of health and illness, and Lipowski’s disease perception concept, this study verified the role of illness perception and perceived stress in existential meaning in Polish HF patients. **Methods**: This manuscript presents the results of a cross-sectional study. Overall, 336 HF patients from Poland were examined. Four questionnaires were used: the Meaning in Life Questionnaire (MLQ), the Multidimensional Existential Meaning Scale (MEMS), the Perceived Stress Scale (PSS-10) and the Disease-Related Appraisals Scale (DRAS). **Results**: Negative illness perception and positive cognitive assessment of the illness were shown to be significant predictors of meaning in life in patients with HF. Furthermore, this relationship was mediated by perceived stress. Additionally, the positive correlation between negative illness assessment and positive illness perception was found. **Conclusions**: This study demonstrates that cognitive assessment of the disease can be associated with the existential resources of heart failure patients. It also highlights the importance of working on the existential sphere of cardiac patients and accurately verified theoretical assumptions regarding the relationship between illness perception and meaning in life, providing a basis for future longitudinal studies and meaning-oriented psychological help focused on individuals with HF.

## 1. Introduction

Heart failure (HF) deserves special attention from a psychocardiological perspective. It is most often caused by cardiovascular diseases that affect the flow of blood into and out of the heart’s ventricles. It occurs when the heart is unable to pump the appropriate amount of blood to meet the body’s needs [1,2]. It most often appears as a result of chronic hypertension and chronic coronary heart disease. From a mental health perspective, depression is the most common psychological cause and also a consequence of heart failure. HF diagnosis is extremely difficult because its symptoms can occur in the course of many other chronic diseases [2,3]. It is an extremely unpredictable disease associated with a large number of hospitalisations, which makes HF a huge existential, psychological and even an economic problem for patients struggling with it [2,4,5].

One of the fundamental problems faced by cardiac patients is trying to answer the question of how to lead a meaningful life despite a cardiac diagnosis. A review of 113 studies (total number of respondents = 21,509) on the relationship between existential resources and cardiovascular diseases indicates the significant role of meaning in the lives of patients with cardiovascular problems [6]. According to Joel Vos, meaning in life is a moderately strong predictor of cardiovascular events and also reveals an important role in the short- and long-term impact of cardiac disease on patients’ psychophysical functioning. Meaning in life resources play a central role in the lives of patients with heart disease, both before and after diagnosis. An aforementioned analysis of 113 articles shows that concerns about the meaning in life can lead to greater stress, reduced motivation to change lifestyle and poorer quality of life and well-being among patients with cardiovascular problems. Joel Vos also notes that the ability to lead a meaningful life after cardiac events is associated with less stress and better mental health in cardiac patients [6].

Studies on individuals with cardiovascular diseases also confirm the relationship between the sense of meaning in life and measures of quality of life and well-being. Park et al. [7] conducted a longitudinal study on a sample of 155 patients with heart failure. They examined the relationship between the sense of meaning in life and measures of health-related quality of life. They found that the sense of meaning in life was positively and moderately associated with measures of health-related physical and psychological quality of life. In the Polish study by Krok and Gerymski on cardiac patients, it was proven that the presence of meaning in life was positively and moderately associated with life satisfaction and positive affect, and negatively and moderately associated with negative affect [8]. The aforementioned study was conducted on a sample of 176 patients diagnosed with coronary heart disease, heart failure, heart defects and arrhythmia. In subsequent analyses by Gerymski and Król [9], the relationship between existential meaning and life satisfaction was examined in 222 Polish heart failure patients with implantable cardioverter-defibrillators (ICDs). It was found that meaning in life was positively and moderately associated with subjective assessment of patients’ life satisfaction.

Finding meaning in one’s life is important in every person’s life, and this issue becomes especially important when a person’s life is at risk [2,10]. According to a meta-analysis cumulating the results of 73,546 participants, the relationship between the sense of meaning in life and physical health is weak to moderate [11]. Existential resources can contribute to improving the subjective assessment of a sense of control, related to self-efficacy, affect and optimism, thus positively impacting one’s health, well-being and quality of life [11]. It is therefore crucial to search for potential predictors that shape the sense of meaning in life in heart failure and other cardiac diseases. Two theoretical models provide guidance: Leventhal et al.’s common-sense model of self-regulation of health and illness [12,13,14] and Lipowski’s disease perception concept [15]. Both theoretical models are relatively consistent and indicate that two potential variables (illness perception and perceived stress) may be important in shaping meaning in life in heart failure patients. Leventhal et al. [12,13,14] demonstrate that the assessment of one’s illness is related to perceived stress and coping. Illness symptoms can contribute to illness perception. Patients may perceive their illness as a threat, which can trigger emotional reactions such as increased ill-being (symptoms of depression, anxiety and perceived stress) in response to these worsening emotional states. They hypothesise that external and internal stimuli can generate a representation of emotional state and health, leading to procedures for coping with stress and evaluating outcomes. Lipowski’s concept [15] also confirms that the assessment of one’s own illness is related to stress and coping styles, and significantly fills a gap in the concept of Leventhal et al. He assumes that a positive assessment of one’s own illness can lead to positive emotional states, which might be responsible for the use of coping methods that favour the treatment process. In turn, a negative perception of the illness can be associated with negative emotional states (such as increased perceived stress), which might be the basis for the use of coping methods that hinder the treatment process [15,16]. Patients who perceive their illness as a threat or punishment will experience existential dilemmas. Furthermore, a negative cognitive assessment of the disease may intensify symptoms of ill-being, such as increased symptoms of depression, increased symptoms of anxiety or perceived stress. It can be assumed that meaning in life and illness perception are significantly correlated in patients with HF. Based on available theoretical models and research results, it can also be theorised that illness appraisal, especially its negative perception, may be related to perceived stress, which can mediate the relationship between cognitive appraisal of the illness and existential resources [2,6,14,16].

The subject of illness perception in heart failure does not seem to be a popular topic among psychocardiology researchers. There is little research on this topic, and most of it is conducted primarily within the paradigm of Leventhal et al.’s model [2,14]. Giardini and colleagues [17] conducted a cross-sectional study on a sample of 120 patients with heart failure. They found that perceived consequences of the disease, symptom experience, emotional reactions and disease-related anxiety were significantly associated with the severity of anxiety and depressive symptoms in their study participants. These results appear to complement the research by Chen et al. [18] on a sample of 302 people diagnosed with heart failure. They demonstrated that depressive symptoms in the study sample were positively correlated with scores regarding the burdensomeness of perceived physical symptoms and the perception of the disease. It is also worth citing the research by Nahlén Bose and her team [19]. They showed that the severity of anxiety in the study sample of 103 patients with heart failure was significantly related to the scores on perceived consequences, treatment control and illness identity. The above-mentioned studies clearly emphasise the role of cognitive assessment of the disease for psychological variables that may shape the existential resources of cardiac patients. Unfortunately, the direct relationships between these variables have not yet been verified.

**Figure 1:**
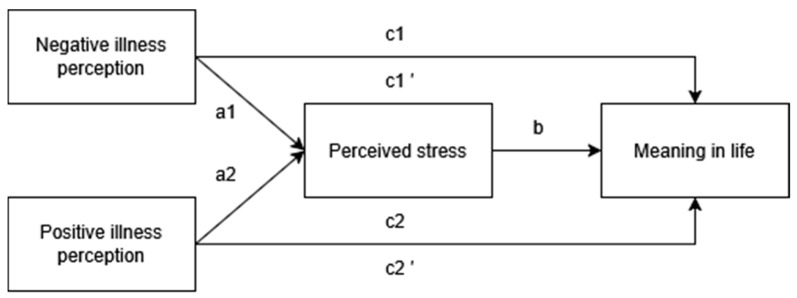
The hypothesised model of the indirect effect of perceived stress on the relationship between illness perception and meaning in life.

It has not yet been studied how the cognitive representation of illness, based on Lipowski’s concept [15], shapes the well-being and meaning in life in heart failure. There is a vast literature discussing the relationship between illness perception and quality of life. However, there is a lack of relevant data verifying the theoretical assumptions regarding the relationship between illness perception and existential resources. Moreover, there are no studies on patients with heart failure that would verify the assumptions of Lipowski’s disease perception concept. Therefore, this project aimed to verify such relationships based on two theoretical models: Leventhal et al.’s common-sense model of self-regulation of health and illness and Lipowski’s illness perception concept. Based on these theories and available research, the following hypotheses were formulated: (H1) negative illness perception will be negatively associated with meaning in life in patients with heart failure; (H2) positive illness perception will be positively associated with the levels of meaning in life in this group of individuals; (H3) the indirect effect of perceived stress in the relationship between illness perception and meaning in life will be significant (because, as the cited theoretical models suggest, the relationship between illness perception and well-being measures is not direct). Figure 1 presents a visualisation of the hypothesised model.

## 2. Materials and Methods

### 2.1. Participants and Procedure

A total of 792 individuals were invited to participate in the study, of which only 355 patients (55.18%) agreed to participate; the most common reason for refusal was fatigue of the respondents. Due to significant data gaps in some completed questionnaires (*n* = 19), this paper presents the results of 336 patients with heart failure. In order to verify if the studied sample is sufficient to test the hypothesised relationships, a Monte Carlo simulation was conducted. The following parameters were used in the simulation: alpha level of 0.05, power (1-Beta) of 0.95, minimum acceptable threshold of CFI = 0.90 and RMSEA = 0.08. The simulation showed that the proposed SEM model can be tested.

Recruitment of participants was conducted at facilities specialising in the treatment of cardiac diseases. Heart failure patients were sought in outpatient clinics, cardiology hospital departments and internal medicine hospital wards; it is common practice in Poland that patients struggling with heart diseases are admitted to internal medicine wards if the hospital does not have a specialised cardiology ward. Cardiac surgery hospital wards were not included in the participants’ recruitment process due to the high invasiveness of treatment in these facilities. Data were collected from 10 Polish clinics and wards. Patients were identified based on available medical records, the opinions of the attending physician, the opinions of nurses and the participants’ own statements. Only adults who were able to provide informed consent to participate in the study, had no cognitive deficits and were not struggling with psychotic disorders were included in the study. These indicators were verified through a short (~15 min) interview with the study participants. Patients were eligible for participation in the study if they had a diagnosis of heart failure, based on medical records and NYHA scale results (category 2 or 3). Individuals with NYHA 1 (no heart failure symptoms) or NYHA 4 (indicating acute heart failure symptoms) were excluded from the study. Patients with NYHA 2 and 3 were grouped together due to methodological issues with this scale.

Empirical studies show that the NYHA scale is not objective. Determining the presence of heart failure symptoms is based on the diagnostician’s individual preferences. An empirical analysis of the evaluations of 30 cardiologists revealed no common method for assigning patients to any of the NYHA classification classes [20]. According to data, the same group of patients was often accidentally assigned to class 2 and class 3. This demonstrates that mild and moderate heart failure symptoms (class 2 and class 3) can be difficult to distinguish, even for a medical professional. Therefore, for the purposes of this study, the NYHA 2 and NYHA 3 patient groups were treated as a homogeneous group.

### 2.2. Measures

*Sociodemographic and biomedical data.* For the purposes of this study, a questionnaire was developed to gather basic information about the study sample. It included questions about the gender, age, education, place of residence, marital status and occupational status of the study patients. It also measured how long the subjects had been struggling with cardiovascular disease, whether they had been hospitalised in the 30 days preceding the study and whether they were under the regular care of a cardiology clinic. It also verified whether they struggled with different cardiac problems or had undergone procedures such as: (1) coronary bypass surgery or coronary angioplasty, and (2) pacemaker (CRT) or cardioverter-defibrillator (ICD) implantation.

*Meaning in Life Questionnaire* [21]. The questionnaire consists of 10 items on a 7-point scale, where 1—“I strongly disagree”; 7—“I strongly agree”. The MLQ is a widely used tool used to measure two dimensions of meaning in life: presence (5 questions) and search (5 questions). Higher scale scores indicate greater levels of a given dimension. In the present study, the scale achieved good reliability indicators (α = 0.79–0.87; ω = 0.79–0.89).

*Multidimensional Existential Meaning Scale* [22]. The original version of the scale includes 15 items measuring three dimensions of meaning in life: comprehension, purpose and mattering. The Polish version of the questionnaire consists of only 9 questions [23] on a 7-point scale, where 1—“I strongly disagree”; 7—“I strongly agree”. A higher score on the scale indicates a greater level of a given dimension. In the presented study, the MEMS scale demonstrated good reliability (α = 0.73–0.90; ω = 0.80–0.91).

*Disease-Related Appraisals Scale* [24]. It is an attempt to synthesise Lipowski’s [15] concept of illness perception and the assessment of the illness in light of Lazarus and Folkman’s [25] transactional stress theory. The scale consists of 47 questions, to which participants respond using a nominal scale (“yes”; “probably yes”; “don’t know”; “probably no”; “no”). The scoring of responses depends on the specific question and differs for non-reversed and reversed questions. After recoding, a higher score on a given subscale indicates a greater intensity of a given type of appraisal. The DRAS measures illness appraisal in light of six categories: threat, benefit, obstacle/loss, challenge, harm and value. The instrument additionally measures one control scale: meaning (importance of a disease). In the present study, the DRAS demonstrated good reliability (α = 0.66–0.87; ω = 0.68–0.87). The lowest reliability coefficients (below 0.70) were achieved only by two subscales: the challenge (α = 0.66; ω = 0.67) and control scale (α = 0.69; ω = 0.69).

*Perceived Stress Scale PSS-10* [26]. Participants respond to 10 questions using a 5-point scale, where 0—“never”; 4—“very often”. Higher scores on the PSS-10 scale indicate higher levels of perceived stress. The questionnaire does not have any subscales. Only a global score is calculated, relating to somatic and mental symptoms of psychological stress. In the present study, the PSS-10 scale demonstrated very good reliability (α = 0.86; ω = 0.87).

### 2.3. Statistical Analysis

Hypotheses were verified based on two types of analyses. First, Pearson’s r correlation analysis was used to verify the assumed relationships. The mediation analysis was performed using the structural equation modelling (SEM) with the maximum likelihood estimator (ML). In addition to the basic measures of χ^2^ and CMIN/df, two measures of absolute fit (SRMR and RMSEA) and two measures of incremental/relative fit (CFI and TLI) were used. All measures of model fit were interpreted based on the values proposed by Hu and Bentler [27]. Acceptable factor loadings were considered to be greater than 0.50 [28]. The reliability and validity of latent constructs were verified based on the CR (construct composite reliability) and AVE (average variance extracted) coefficients. The following values were considered to indicate good psychometric properties of the constructs: CR > 0.70, AVE > 0.50, and √AVE greater than the correlation between the constructs [28]. The tested indirect effect was verified using the BC (bias-corrected) bootstrapping method with 5000 samples [29]. All described statistical analyses were performed using IBM SPSS Statistics 23 with the AMOS add-on [30].

## 3. Results

### 3.1. Sociodemographic Characteristics of the Studied Sample

This paper presents the results of 336 patients with heart failure. The sample included 194 women (57.74%) and 142 men (42.26%). The age of the participants ranged from 18 to 92 years (M = 54.99; SD = 17.27). The mean duration of the disease in the study sample was ~11 years, but it had a relatively large standard deviation (M = 11.05; SD = 10.36), indicating that the study included patients with varying degrees of experience with the HF. Only 38 patients (11.31%) reported being hospitalised within the 30 days preceding the study. Moreover, 308 individuals confirmed being under the regular care of a cardiology clinic (91.67%). All participants had heart failure consistent with the NYHA 2 or 3 class. Only 45 patients reported having a CRT or ICD. The study also used a measure described as “Number of cardiac diagnoses,” which is the sum of the number of cardiac diagnoses reported by the participants (M = 2.66; SD = 2.33). Most often, patients declared that they were struggling with hypertension (primary or secondary) and ischemic heart disease (including coronary artery disease)—the two most important predictors of HF development.

### 3.2. Relationships Between Studied Variables

Table 1 presents the correlations between the tested variables. For clarity, statistically non-significant relationships have been removed. From the sociodemographic and biomedical data, only age was associated with selected psychological variables: perceived stress (negative and weak correlation) and the presence of meaning in life (positive and weak correlation). Illness duration and number of cardiac diagnoses were not associated with any variable. Polynomial (non-linear) effects of those two were also not statistically significant.

A significant relationship was observed between illness perception and perceived stress. Negative illness perception was positively and moderately associated with stress levels. Positive cognitive assessment of one’s illness was negatively and weakly associated with stress (the exception being the perception of illness as a benefit).

Significant correlations were also observed between illness perception and perceived stress with existential resources. Perceived stress was negatively and moderately associated with the resources of meaning in life. Negative illness perception was negatively and weakly-moderately associated with meaning in life. Positive cognitive appraisal of illness was positively and weakly-moderately correlated with the levels of meaning in life. The exception is search for meaning in life, which is theoretically consistent—this variable was positively and weakly associated with the level of perceived stress and negative illness perception (the worse the illness appraisal and perceived stress are, the greater the intensity of the search for meaning in life is).

It should be underlined that the negative perception of the disease was more strongly associated with the levels of meaning in life than the positive cognitive assessment of one’s health condition.

### 3.3. Indirect Effect Analysis Using SEM

Next, SEM analysis was used to verify the indirect effect of perceived stress. The hypothesised model achieved an acceptable model fit. One non-significant path was found—search for meaning in life was not a good predictor of the latent variable encompassing existential resources, and it presented a factor loading of −0.08. Therefore, it was decided to remove it. The final model had an acceptable fit. No modification indices were used in the model, and no error covariances were established. The obtained measures of model reliability and validity were also acceptable, allowing us to consider the model worthy of further analysis and interpretation. Table 2 presents the detailed data from the analysis.

**Figure 2:**
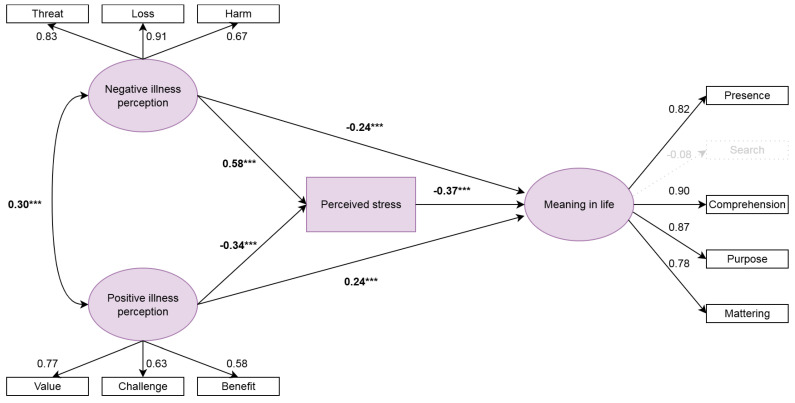
Final model obtained in SEM. No modification indices or error covariances were used. The observed variable that was not included in the final model is presented in a transparent form. Note: *** *p* < 0.001.

The SEM model confirmed the theoretical assumptions. Negative illness perception and perceived stress were negative predictors of meaning in life. Positive cognitive evaluation of illness was a positive predictor of existential meaning. Interestingly, negative and positive illness perceptions were positively related. Also, the indirect effect of perceived stress in the relationship between illness perception and meaning in life was significant (see Table 2). Figure 2 presents a visualisation of the final model. The observed variable that was not included in the final model (search for meaning) is presented in a transparent form. The model explained a large percentage of the variance in the dependent variable scores (see Table 2 for R-squared and Cohen’s f-squared coefficient values).

Principal Component Analysis (PCA) revealed that the single factor explained 19% of the total variance. This value is well below the commonly accepted threshold of 50%, which could indicate a significant common method bias problem. Then, the comparison of the base model and the model including the Common Latent Factor (CLF) was performed. The standardized regression coefficients obtained in both models were then compared. The differences between the coefficients were small, ranging from 0.018 to 0.134. None of the analysed relationships showed significant changes in interpretation after including the method factor. Therefore, the results suggest that common method variance does not pose a significant threat to the obtained results. Lastly, the Variance Inflation Factor (VIF) coefficients were calculated to exclude the occurrence of collinearity. The VIF coefficient value for perceived stress was equal to 1.488, and for the meaning in life, it was 1.499—this means that multicollinearity was not observed in the presented data.

## 4. Discussion

The study presented the results of 336 participants. The formulated hypotheses were confirmed. Based on two theoretical models (Leventhal et al.’s common-sense model of self-regulation of health and illness and Lipowski’s illness perception concept), it was positively verified that (1) negative illness perception was negatively associated with meaning in life in patients with heart failure; (2) positive illness perception was be positively associated with the levels of meaning in life in this group of individuals; and (3) the indirect effect of perceived stress in the relationship between illness perception and meaning in life was significant.

The relationships between illness perception and meaning in life are consistent with the presented theory. They do not require extensive discussion. It is obvious that a negative perception of illness can cause (both directly and indirectly) existential dilemmas concerning the meaning of life, thoughts about death or a sense of coherence in one’s health. Perceived stress was observed to possibly mediate this relationship. This demonstrates that general stress levels, not just illness-related distress, may play a significant role in the relationship between cognitive appraisal of illness and resources for meaning in life. This shows the need to take care of the mental sphere of patients not only in the area of their illness, but also in their entire functioning in other areas of life.

This suggests that we should look for factors that can affect the relationship between stress and meaning in life in heart failure patients. Coping is an obvious one. However, the role of personal and relational resources is also worth examining. Cohen and Wills’ [31] buffering hypothesis is one of the most popular theoretical models examining the role of social support in the relationship between stressful life events (such as illness) and well-being. It posits that social support serves as a buffer between perceived stress and well-being. It can mitigate the impact of unpleasant and burdensome experiences and negative emotions on an individual’s functioning. Cohen and Wills’ [31] model assumes that the correlation between stress and health is weaker when an individual has a high level of social support. For this reason, future projects should also examine the role of support in the functioning of patients diagnosed with HF. On the other hand, according to Antonovsky’s concept of salutogenesis [32,33], health and well-being depend on generalised resistant resources (GRRs), stressors, lifestyle and personal resources such as a sense of coherence. GRRs facilitate effective coping with everyday stressors and perceiving the world as meaningful. Based on the salutogenetic model, it should be recognised that many personal resources may play a significant role in the relationship between illness perception and meaning in life in patients with HF. These include a sense of coherence, self-efficacy, resilience, hardiness or even self-esteem.

In the presented study, a positive correlation was also observed between negative illness perception and positive cognitive assessment of one’s health status. This may be related to the process of adaptation to illness. Positive emotions associated with illness may coexist with negative ones. The presented correlation may suggest that patients engage in a process of meaning-making and re-evaluation of their own state [34]. To find “value” in illness, patients must first recognise it as something significant (often negative). Without a sense of the burden of illness, there is no need to search for deeper meaning. Therefore, this positive relationship is not surprising. This can be supported by a longitudinal study on patients after percutaneous coronary intervention for coronary heart disease, emphasising that the severity of ill-being was a significant covariate of the relationship between optimism and the quality of life of these patients [35]. This is also consistent with data on post-traumatic growth (PTG) in cardiac patients, which clearly demonstrate that negative perception of the condition can be a basis for creating a new, positive hierarchy of values and positive perception [36,37].

The presented results can serve as a guide for determining the impact of psychological interventions on individuals with heart failure. Analysing the data from meta-analyses, it should be noted that psychological support can positively impact the functioning and implementation of positive changes in the lives of patients with heart failure, but this requires good mental functioning [38]. In their literature review, Mierzyńska, Jurczak and Piotrowicz [39] emphasise that the use of solution-focused therapy is positively associated with patients’ coping with the effects of cardiac disease and motivational processes, which has a positive impact on their functioning and health. Also, meaning-oriented psychological support and psychotherapy can be effective. Heart failure patients sometimes struggle with unjustified thoughts known as cognitive distortions. These cognitive biases can take the shape of thoughts such as: “No matter what I do, I won’t get better” (arbitrary reasoning), or “There’s no point in trying, because I’ll die from heart disease anyway” (catastrophizing) [2]. Perceiving one’s life as meaningless and purposeless, perceiving illness as a punishment for sins, an insurmountable obstacle to achieving goals or assessing it as an impossible state to change (i.e., regain health) are examples of irrational thoughts that can be ruminative in nature. For this reason, cognitive-behavioural therapy techniques appear to be particularly valuable in working with patients struggling with the stress of heart failure. The use of CBT in working with cardiac patients is also supported by empirical data. Meta-analyses by Helal et al. [40] and Chernoff et al. [41] demonstrated the effectiveness of CBT in reducing the risk of hospitalisation in heart failure patients and improving their mental functioning. As mentioned in the introduction, one of the fundamental problems faced by cardiac patients is trying to answer the question of how to lead a meaningful life despite a cardiac diagnosis [6]. The presented study shows that interventions aimed at improving the existential sphere of HF patients should also take into account aspects of illness perception, perceived stress and coping.

The presented study is the first to explain how cognitive representation of illness, based on Lipowski’s concept [15], shapes the meaning in life in individuals with heart failure. It provides important results for further cross-sectional and longitudinal studies as well as therapeutic interventions. Nevertheless, it has some limitations. The lack of correlation between sociodemographic and biomedical data and the tested variables may be due to the nature of the study sample, as seen in the values of the duration of illness, age and number of cardiac diagnoses reported by the patients (their mean and standard deviation). The study included individuals with HF with a very broad range of illness experience. The diversity of the sample, which shows that it was homogeneous in the case of some variables, makes it difficult to create general recommendations. Unfortunately, the size of the study population did not allow for subgroup division. Future studies would be beneficial by targeting specific patient groups and conducting analyses, for example: (1) comparing young adults to older adults, (2) comparing individuals with acute heart failure to those with less severe illness and (3) comparing individuals with very short illness experience to those with long-term chronic illness. Also, this study did not examine many factors relevant to the well-being and ill-being of HF patients, such as mental health, social support, economic status or personality traits. This limits the generalizability of the results because, as mentioned, the participants presented a wide range of HF experiences. Another significant limitation is the lack of measurement of patient parameters such as blood pressure or LVEF (left ventricular ejection fraction) levels. This is due to the nature of the study. Psychologists often do not have access to such information. Poor parameters will shape the variables tested in this study. This necessitates conducting psychocardiological research in collaboration with specialists in both medicine and mental health. Moreover, the study is cross-sectional. This is both a disadvantage and an advantage. On the one hand, it does not allow for discussion of the influence between variables and does not verify the stability of relationships over time. Given the cross-sectional nature of the study, alternative models (e.g., meaning in life affecting perceived stress) cannot be excluded. Therefore, the proposed model should be regarded as one of several plausible relationships—in this case, very well grounded in the theoretical models suggested by Lipowski and Leventhal. On the other hand, from an economic and patients’ perspective, it allows for obtaining results for further research without the exploitation of financial resources and burdening patients. Pilot studies in clinical groups are important, at least from a psychological perspective—it is ethical not to overwhelm patients with exploratory studies, which are precisely what longitudinal studies are. Therefore, the described study can be considered a good pilot study for more engaging projects.

## 5. Conclusions

This study demonstrates that cognitive assessment of the disease can be important for the existential resources of heart failure patients. It also highlights the importance of working on the existential sphere of cardiac patients and accurately verifies theoretical assumptions regarding the relationship between illness perception and meaning in life, providing a basis for future longitudinal studies. The next step would be to verify the tested model in a longitudinal study and evaluate the effectiveness of therapeutic interventions on HF patients’ well-being, ill-being and quality of life based on meaning-oriented psychoeducation and support.

## Figures and Tables

**Table 1:** Relationships between studied variables (N = 336).

	Perceived Stress	Meaning in Life				
		Presence	Search	Comprehension	Purpose	Mattering
Perceived stress	-	−0.50 ***	0.11 *	−0.45 ***	−0.44 ***	−0.42 ***
Age	−0.22 *	0.13 *	-	-	-	-
Illness duration (in years)	-	-	-	-	-	-
Number of cardiac diagnoses	-	-	-	-	-	-
Illness perception: Importance (control)	0.38 ***	−0.18 ***	-	−0.14 **	−0.12 *	-
Illness perception: Threat	0.40 ***	−0.22 ***	0.14 *	−0.24 ***	−0.23 ***	−0.22 ***
Illness perception: Obstacle/loss	0.42 ***	−0.28 ***	0.13 *	−0.26 ***	−0.29 ***	−0.28 ***
Illness perception: Harm	0.33 ***	−0.26 ***	0.17 **	−0.19 ***	−0.20 ***	−0.19 ***
Illness perception: Benefit	-	-	-	-	-	-
Illness perception: Challenge	−0.11 *	0.24 ***	-	0.20 ***	0.24 ***	0.17 **
Illness perception: Value	−0.17 *	0.20 ***	0.12 *	0.13 *	0.13 *	0.15 **

Note: * *p* < 0.050; ** *p* < 0.010; *** *p* < 0.001; statistically non-significant results have been removed from the table to improve its clarity.

**Table 2:** Results of the structural equation modelling (SEM; N = 336).

**Model Fit**				**χ^2^**	***p***	**CMIN/df**	**CFI**	**TLI**	**SRMR**	**RMSEA** **[LLCI; ULCI]**
Hypothesized model				163.222	<0.001	3.331	0.938	0.916	0.068	0.083 [0.069; 0.098]
Final model				139.567	<0.001	3.579	0.945	0.922	0.063	0.088 [0.072; 0.104]
Δ—hypothesised vs. final				23.655	0.008	0.248	0.007	0.006	0.005	0.005
**Reliability and validity: CR, AVE & √AVE**						**CR**	**AVE**	**1.**	**2.**	**3.**
Negative illness perception (Negative IP)						0.848	0.654	0.809	-	
2. Positive illness perception (Positive IP)						0.701	0.442	0.300	0.665	-
3. Meaning in life (MIL)						0.833	0.568	−0.344	0.223	0.754
**Direct effects (a, b, c′)**					**b**	**SE**	**β**	**R^2^/Cohen’s f^2^**	**LLCI**	**ULCI**
Negative IP	→		Stress		0.672	0.068	0.575	0.328/0.488	0.552	0.794
Positive IP	→		Stress		−0.818	0.162	−0.342		−1.185	−0.516
Negative IP	→		MIL		−0.206	0.061	−0.240	0.333/0.499	−0.322	−0.097
Positive IP	→		MIL		0.416	0.126	0.236		0.178	0.718
Stress	→		MIL		−0.276	0.048	−0.375		−0.363	−0.192
**Indirect effects (a × b)**					**b**	**SE**	**β**	**LLCI**	**ULCI**	
Negative IP	→	Stress	→	MIL	−0.185	0.040	−0.216	−0.289	−0.154	
Positive IP	→	Stress	→	MIL	0.226	0.032	0.128	0.081	0.187	
**Total effects (c)**					**b**	**SE**	**β**	**LLCI**	**ULCI**	
Negative IP	→		MIL		−0.391	0.068	−0.455	−0.568	−0.344	
Positive IP	→		MIL		0.642	0.077	0.364	0.234	0.490	

Note: AVE coefficient for positive illness perception is lower than 0.50 due to the number of observed variables and their factor loadings. Based on the Fornell-Larcker criterion (AVE lower than 0.50 but CR higher than 0.60), it can be assumed that this latent variable shows acceptable psychometric properties; The direction of the regression path is represented by the symbol →.

## Data Availability

The data can be made available from the corresponding author upon reasonable request.
